# Stem cells, senescence, neosis and self-renewal in cancer

**DOI:** 10.1186/1475-2867-6-25

**Published:** 2006-11-08

**Authors:** Rengaswami Rajaraman, Duane L Guernsey, Murali M Rajaraman, Selva R Rajaraman

**Affiliations:** 1 Department of Medicine, Division of Hematology, Dalhousie University, Halifax NS. B3H 1X5; 2 Department of Pathology, Dalhousie University, Halifax NS. B3H 1X5, Canada; 3 Nova Scotia Cancer Centre, Department of Radiation Oncology, QEII Health Sciences Center, Dalhousie University, Halifax NS. B3H 1X5, Canada; 4 Downtown Clinic, Windsor, ON, N9A 1G5, Canada

## Abstract

We describe the basic tenets of the current concepts of cancer biology, and review the recent advances on the suppressor role of senescence in tumor growth and the breakdown of this barrier during the origin of tumor growth. Senescence phenotype can be induced by (1) telomere attrition-induced senescence at the end of the cellular mitotic life span (MLS*) and (2) also by replication history-independent, accelerated senescence due to inadvertent activation of oncogenes or by exposure of cells to genotoxins. Tumor suppressor genes p53/pRB/p16INK4A and related senescence checkpoints are involved in effecting the onset of senescence. However, senescence as a tumor suppressor mechanism is a leaky process and senescent cells with mutations or epimutations in these genes escape mitotic catastrophe-induced cell death by becoming polyploid cells. These polyploid giant cells, before they die, give rise to several cells with viable genomes via nuclear budding and asymmetric cytokinesis. This mode of cell division has been termed neosis and the immediate neotic offspring the Raju cells. The latter inherit genomic instability and transiently display stem cell properties in that they differentiate into tumor cells and display extended, but, limited MLS, at the end of which they enter senescent phase and can undergo secondary/tertiary neosis to produce the next generation of Raju cells. Neosis is repeated several times during tumor growth in a non-synchronized fashion, is the mode of origin of resistant tumor growth and contributes to tumor cell heterogeneity and continuity. The main event during neosis appears to be the production of mitotically viable daughter genome after epigenetic modulation from the non-viable polyploid genome of neosis mother cell (NMC). This leads to the growth of resistant tumor cells. Since during neosis, spindle checkpoint is not activated, this may give rise to aneuploidy. Thus, tumor cells also are destined to die due to senescence, but may escape senescence due to mutations or epimutations in the senescent checkpoint pathway. A historical review of neosis-like events is presented and implications of neosis in relation to the current dogmas of cancer biology are discussed. Genesis and repetitive re-genesis of Raju cells with transient "stemness" via neosis are of vital importance to the origin and continuous growth of tumors, a process that appears to be common to all types of tumors. We suggest that unlike current anti-mitotic therapy of cancers, anti-neotic therapy would not cause undesirable side effects. We propose a rational hypothesis for the origin and progression of tumors in which neosis plays a major role in the multistep carcinogenesis in different types of cancers. We define cancers as a single disease of uncontrolled neosis due to failure of senescent checkpoint controls.

## Background

Mitosis and meiosis are the classic modes of cell division, which have engaged the attention of the scientists for more than a century. While mitosis involves the symmetric division of a diploid, somatic cell to yield two diploid daughter cells identical to the mother cell, meiosis or reduction division yields varying number of haploid daughter cells with chromosomes carrying new combinations of alleles due to the cross-over phenomena. While the former is responsible for the somatic growth of multicellular organisms from the single celled zygote, the later is involved in sexual reproduction by producing oocytes or sperms, which will reconstitute the diploid somatic cells after fertilization. In both cases, the nuclear envelope is disassembled to facilitate accurate chromosome distribution during karyokinesis, and is reassembled at the end of kayrokinesis during the telophase [1]. Errors in chromosome segregation during mitosis may cause aneuploidy, which will be detrimental to the cell and if viable, the cell will gain genomic instability, possibly leading to cancer growth. In order to assure that the somatic cells faithfully duplicate and distribute the genomic DNA to daughter cells with high fidelity, several checkpoint controls have evolved to regulate the mitotic cell cycle progression in order to maintain genomic stability in daughter cells [Reviewed in [2-4]]. In addition, cells have evolved tumor suppressor program consisting of tumor suppressor genes and apoptosis genes.

Recently, the role of senescence as a tumor suppressor program has been demonstrated both in vitro and in vivo, which is considered to be as efficient as the apoptosis program. Cellular senescence can set in under different circumstances, which fall into two major categories: (1) replication history-dependent telomere attrition-induced senescence and (2) replication history-independent senescence. Senescence phenotype induced by exposure to chemotherapeutic agents fall in the second category. These agents induce accelerated senescence in both normal and tumor cells. Cellular events linking accelerated senescence to mitotic arrest and cell death via mitotic catastrophe, and eventual out growth of aneuploid neoplastic cells from such senescent populations have not been clearly understood. In this review, (1) we summarize the current concepts of cancer self-renewal, (2) draw attention to recent developments in the understanding of the role of senescence in cancer and (3) describe the novel mode of cell division termed **neosis**, which helps cells bypass senescence and aids tumor growth [5,6]. Further, we discuss the implications of these findings to the current concepts of cancer biology and propose a neosis-based multistep carcinogenesis hypothesis that provides a more rational explanation of the steps involved in the origin and progression of tumors.

### Current concepts in cancer biology

The essentials of the current concepts of cancer growth are three fold: (1) Cancer originates via mitotic division of normal cells with DNA damage [7]; (2) Cancer cells originate from mutant stem cells called cancer stem cells (CSCs), which have the potential to self-propagate by asymmetric division yielding one CSC and one differentiating into tissue-specific tumor cell; [8-14] and (3) since CSCs retain the properties of stem cells, they are immortal and have unlimited division potential without being subjected to the phenomenon of aging. Emerging new evidence hints at a major revision of these current concepts about cancer.

### Mitosis and cancer

Current concept of cancer is based on the belief that tumor cells arise after about 13 mitotic divisions of the initiated cell [7]. In addition, cancer cells also multiply by mitotic division. Therefore, the conventional non-surgical cancer treatments, consisting of chemotherapy and radiotherapy target only the mitotic populations of tumor cells. They do not differentiate between proliferating normal and tumor cells. This results in undesirable side effects that limit the level of usable dose-intensity of these treatments and restrict their application to only the fittest of patients, this approach results in the growth of resistant tumor cells in the place of originally responsive tumor often in a matter of months by a mechanism that has not been clearly understood. Development of novel strategies to improve current status of cancer therapy will require identification and exploitation of yet unrecognized differences between normal and tumor cells with respect to propagation, evolution and development of resistance to conventional treatments [15,16].

### Immortality of cancer cells and limited mitotic life span of diploid somatic cells

The common belief that cancer cells are immortal probably originated from the early attempts at mammalian cell culture *in vitro*. Gey et al. [17] grew normal epithelial cells and also cultured cells from invasive colon carcinoma. They established one of the oldest cancer cell cultures, HeLa cells with continuous division potential. This resulted in the notion that cells are immortal, while the whole organisms were mortal.

With the advent of sterile culture conditions and improved culture media, Hayflick published his well known study on normal embryonic diploid human lung fibroblasts. Hayflick suggested that there was a "finite limit to the cultivation period of diploid cell strains" and that this was "attributable to the intrinsic factors which are expressed as senescence at the cellular level," while transformed or cancer cells were capable of unlimited mitotic division potential and, therefore, were considered 'immortal' [18]. It has been now well established that this Hayflick limit and the phenomenon of senescence are due to the limited mitotic division potential of normal human somatic cells. At the end of their limited mitotic life span (MLS), normal cells enter a permanent non-proliferative senescence phase, characterized by a large, flat morphology, a high frequency of nuclear abnormalities and often consisted of multinucleate and/or polyploid giant cells (MN/PG cells). Senescent cells display positive stain for senescence associated β-galactosidase at pH 6.0 (SA-β-gal) [19]. Such cells display mitotic crisis and are thought to die by mitotic catastrophe [20]. Onset of replicative senescence in human cells is caused by the exhaustion of mitotic potential due to telomere attrition as a function of aging [21-23]. Telomere shortening beyond a certain limit triggers DNA damage response [24-26].

### Stem cells and their asymmetric division potential

Stem cells are characterized by (1) the potential to undergo continuous self-renewal and extensive proliferation, (2) the maintenance of a constant pool of the undifferentiated stem cells through the life time of the host and (3) the potential to undergo differentiation into multiple cell types upon receiving the appropriate stimulus from the microenvironment. The primordial stem cell is the fertilized oocyte or zygote, which gives rise to embryonic, germinal and adult stem cells during development. Embryonic stem cells (ESCs) isolated from the inner cell mass of 5–6 day old mammalian embryos are able to differentiate into different cell types derived from all three germ layers and are, therefore, considered pluripotent; ESCs undergo symmetric and logarithmic expansion by mitosis, both daughter cells being identical and retaining pluripotency. Germinal stem cells (GSCs) are confined to the gonads. After mitotic amplification, followed by a terminal meiotic division, they produce the egg or the sperm for sexual reproduction. During the early embryonic development, as the germ layers are formed from the ESCs, the process of cellular determination and organogenesis are initiated. The stem cells begin to undergo asymmetric mitotic division, where one daughter cell maintains the stem cell pool specific for different organs and tissues, and the other starts the process of differentiation through transit amplifying stages and becomes the progenitor of somatic cells. These are the adult stem cells (ASCs) or resident tissue-specific stem cells that are involved in tissue homeostasis and are capable of tissue regeneration. Thus, during development, the ESCs eventually give rise to about 200 different cell types of the adult organism, as would be expected of their pluripotency [6,27].

### Stem cells and Cancer Stem Cells (CSCs)

Studies on the susceptibility of fish and mammalian embryos to tumorigenecity by carcinogens showed that early embryos *in vivo *were resistant to carcinogenic agents. They became increasingly susceptible to tumorigenic mutations by carcinogens, after the development of tissue specific adult stem cells or ASCs and the process of organogenesis was initiated [28-31] The following two different conclusions can be drawn from these data [6]:

(1) Adult stem cells were prone to tumorigenic mutations, while the transit amplifying and differentiating cells were not and the embryonic stem cells were also resistant to tumorigenic mutations due to their innate nature;

(2) Determined adult stem cells (ASCs) and their committed transit amplifying cells and derivatives on the way to differentiation were highly susceptible to tumor initiating mutations, while the embryonic stem cells were relatively resistant.

By this time, the Hayflick's concept of normal somatic cells had limited mitotic division potential [18] and the concept of 'immortality' of tumor cells [17] were well known. It was also demonstrated that stem cells with some mutation might play a role in the clonal origin of some cancers [30-32]. The concept of stem cell origin of cancers was postulated during the later part of the 19^th ^century [14]. The first experimental proof was published by Till and McCullach [33]. Based on teratocarcinoma studies, Pierce [34] proposed that cancer was due to maturation arrest of stem cells. Thus, the scientific atmosphere was ripe to interpret these data on embryonic carcinogenesis induction [30,31,35] to mean that stem cells (ASCs) in regenerating tissues could be susceptible to epigenetic changes, while they still retained their unlimited division potential and, therefore, immortality. The proposal that transformation of normal cells into neoplastic cells with unlimited mitotic division potential was due to genetic and epigenetic alterations in ASCs, with the retention of their unlimited division potential [30,31,35] seemed a reasonable assumption. Thus, the suggestion that mutant ASCs formed during the regenerative stages gave rise to mutant stem cells, and have retained their unlimited division potential was generally accepted. Based on the striking similarities between the ASCs and cancer cells, the 'immortality' of cancer cells has been attributed to a minority of tumor cells that can not only self-renew but also can give rise to a full fledged tumor tissue consisting of stem-like cells and abnormally differentiated cells, thus resulting in the hierarchical nature of tumor cell population [36,37]. These events lead to the formation of a cell mass consisting of a minor percentage of cancer stem cells, proliferating progenitor tumor cells and non-dividing, incompletely differentiated tumor cells making up the bulk of the tumor tissue. Thus, the concept that cancer is a disease of unlimited mitotic division has gained acceptance and the tumor-initiating cancer cells with self-renewal potential have been termed "Cancer Stem Cells" (CSCs). Therefore, akin to the ASCs, CSCs are thought to constitute a constant pool of mutated adult stem cells, and by virtue of their asymmetric division potential, are responsible for the continuous growth of tumor tissue, that consists of CSCs and differentiated tumor cells [8,13,14,37].

The concept that resident adult tissue stem cells turn into CSCs with their own specific surface markers has gained wide acceptance. Stem cell origin of cancers has been recognized in hematopoietic malignancies decades ago [8,14,33,38,39] and more recently in solid tumors such as breast tumors, brain tumors [9-13], melanoma [40] and prostate cancer [41], each having its own specific surface markers. The identification of such CSCs as a target for anti-tumor therapy is currently being actively investigated with the aim of using them as specific targets for cancer therapy [11-13]. However, this concept is still controversial and implies, but has not yet been unequivocally proven, that CSCs are capable of asymmetric division, i.e., one daughter cell maintaining the CSC pool and the other differentiating into tumor cells [6,42-44]. Recently, the theoretical and technical difficulties of the CSC hypothesis have been reported [42]. In addition, it is likely that such tissue specific CSC markers are not unique to CSCs, but may also be shared by normal stem cells and, probably, to a lesser degree by their transit amplifying somatic cell intermediates. Further, since the CSCs are thought to be immortal, this, in turn, implies that they are not subject to aging and senescence, which will inevitably lead to telomere attrition due to senescence-checkpoint and rejuvenation via neosis to turn cancerous (6, Also see below).

### Stem cells (ASCs) resemble somatic cells

Weismann [45] popularized the concept of a complete separation in metazoans between a potentially immortal germ line and a mortal soma that transfers the germ line to the next generation and then senesces. This concept has led to the conviction that somatic cells are subject to senescence, while the germ cells are not. However, it should be pointed out that even the germ cells are also subject to aging along with its host [46]; however, they are rejuvenated by sexual reproduction at the beginning of each generation. According to this definition, adult stem cells would also be considered to be somatic cells, since these are probably committed to differentiate, although they might be more primitive compared to transit amplifying cells [6,27].

In spite of the obvious difference in the division potentials between ASCs (potential to self-renew and unlimited MLS) and differentiating somatic cells (limited MLS), it is becoming increasingly clear that ASCs may lose division potential as they approach their differentiated state, and share several properties of the transit amplifying and differentiating cells. These include: (1) responsiveness of ASCs to both intracellular and extracellular factors to stop cycling and enter differentiation pathways or to enter cycling state and play a role in tissue homeostasis [27,47]; (2) an age-dependent decrease of telomere length in human hematopoietic stem cells (hHSCs), probably contributing to a reduction in their proliferation potential [48,49]; (3) limited division potential of adult human mesenchymal stem cells (hMSCs) *in vitro *that can differentiate into multiple cell lineages including bone, cartilage, adipose and muscle tissues, due to a lack of telomerase [50-52]; (4) the loss of tumor suppressor function of p16^Ink4a^/p14^Arf ^(or senescence checkpoint controls) confers 'immortality' to hMSCs; (5) extension of population doubling of hMSC by transduction of the telomerase gene hTERT *in vitro*; and (6) the emergence of some sublines of the cells with extended MLS, loss of contact inhibition, anchorage independent growth potential and formation of mesenchymal tumors in 10/10 mice by acquired loss of p16^Ink4a^/p21, due to epigenetic inactivation by methylation of DBCCRI gene purportedly involved in the onset of senescence [53]

Conditional telomerase expression caused proliferation of hair follicle stem cells *in vivo *[54], while overexpression of mTERT in basal keratinocytes resulted in increased epidermal tumors and increased wound healing in transgenic mice *in vivo *[55]. These events are similar to the events after telomerase transduction in non-stem somatic cell populations, respectively [6,21-23,48,56-63]. In addition, telomerase is required for retarding the shortening of telomeres and to extend the replicative life span of HSCs during serial transplantation [64]. Expression of hTERT in HIV-specific CD8+ T cells showed both an enhanced and sustained capacity to inhibit HIV-1 replication and enhanced antiviral functions accompanied by an increase in proliferative potential and telomere length stabilization [65]. As in the case of somatic differentiating cells, stem cells also undergo telomere attrition during aging, which is followed by breakage-fusion-bridge cycle, giving rise to self-propagating mechanism for increasing the level of genomic instability via aneuploidy [48,52,63,65-67].

Stem cells are attractive candidates to initiate cancer due to their pre-existing capacity for self-renewal and 'unlimited' proliferative potential, and their likelihood of accumulating mutations through the age of the host [14,47]. However, cancer causing mutations also arise in the more committed progenitors, the mitotic derivatives of stem cells at any state in the differentiation pathways in various tissue cell types [8,68,69]. In fact, most of the *in vitro *studies since 1970s on cancer initiation, promotion, and progression have been carried out not in stem cells, but in differentiating somatic, non-stem cells such as fibroblasts, epithelial cells, myoblasts, chondrocytes etc., of various mammalian species including mouse, rat, hamsters and humans; and the resultant transformed cells isolated using the transformed focus assay do form tumors in the appropriate hosts [5,70-73]. Consistent with the concept of origin of tumor growth from the differentiating population beyond the initiation of determination stage is the fact that almost ~200 different types of human cancers are known, just as many as the number of differentiated cell types [27]. This correlates well with ~200 different microRNAs that are known to be involved in the process of differentiation [74-76]. Recent findings that the microRNA profiles of cancer cells reflect the developmental lineage and differentiation stage of tumors [77] suggest that ASCs and the transit amplifying cells and not simply the 'immortal' ASCs alone are prone to carcinogenic mutations. Even differentiated cells may be prone to turn carcinogenic, when infected with tumor viruses such as acute transforming viruses (Eg. RSV), Human T cell leukemia virus, SV40, human papilloma viruses HPV-5, -8, -16, -18 and -31, human hepatitis virus B, and Burkitt's lymphoma virus etc., since these viruses carry their own transforming genes, that have potential to inactivate the tumor suppressor genes required for the onset of senescence and often carry growth promoting genes [78].

### Senescence as a tumor suppressor mechanism

Since senescent cells do not respond to growth factors, and display terminal mitotic crisis, the phenomenon of senescence is thought to constitute a tumor suppressor program [79-81] and is considered equivalent to the programmed cell death by apoptosis [82]. The relevance of senescence as a tumor suppressor mechanism has been recently demonstrated unequivocally in different tumor systems *in vitro *and *in vivo *[81,83] and constitutes a fail-safe, although not perfect, mechanism to inhibit cancer growth.

While senescent cells do not enter the mitotic cycle even in the presence of growth factors, they are alive and remain metabolically active in culture for several years [84]. This cell cycle arrest is due to the tumor suppressor action of the genes involved in the p53/pRb/p16Ink4 pathway collectively termed the senescence checkpoint control. Abrogation of this pathway by mutation, epigenetic mechanisms or viral inactivation of any one of these genes bypasses senescence checkpoint and the cells grow beyond their normal intrinsic MLS. After an additional 20 – 30 population doublings, the cells enter a terminal mitotic crisis. During this second mitotic crisis period the cells display apoptotic death due to gross chromosomal abnormalities, some rare cells continue to proliferate yielding an 'immortal' cell line, by an unknown mechanism. These two mitotic crisis phases in the normal cell life span have been termed M1 and M2 [58,85].

Accelerated premature senescence in the absence of telomere attrition can be induced by different non-lethal conditions that cause acute genetic duress in primary cells *in vitro *and *in vivo*. Such cells also display similar characteristics such as non-responsiveness to growth factors, large, flat cell morphology with nuclear abnormalities, and stain positive for SA-β-gal and senescence associated heterochromatin formation (SAHF) [Reviewed in [19,84]]. DNA-damage induced repair response is activated in such cells, just as in the case of telomere attrition-induced senescent cells. Such acute genetic stress-inducing factors include unfavorable culture conditions [86], or the addition of aberrant oncogenic and mitogenic signals such as activated H-RAS [87]. In addition, as can be expected, chemotherapeutic agents that induce DNA double strand breaks [88], and mitotic spindle toxins [89,90] are also known to effect accelerated senescent phenotype.

Oncogene-induced premature senescence will terminate a pre-malignant condition before a fully transformed cell can develop from primary cells [80,91] and is associated with the p53, p16INK4a, pRB pathway. Using a mouse model, in which the oncogene *Ras *was activated in the hematopoietic cells of the bone marrow, Braig et al. [92] have demonstrated that cellular senescence phenotype can efficiently block the development of lymphoma. pRB-mediated silencing of growth promoting genes by SAHF was shown to be formed via methylation of histone H3 lysine 9 (H3K9me) [93]. The histone methyltransfearse Suv39h1 protein methylates histones and physically binds with pRB tumor suppressor protein [94,95]. Suv39h1 was shown to be required for oncogene-induced premature senescence due to the introduction of *Ras *in lymphocytes [92]. Proliferation of primary lymphocytes was stalled by a SuV39h1-dependent H3Kme-related senescent growth arrest in response to oncogenic Ras, resulting in the inhibition of the initial step of lymphomagenesis. In Suv39h1-deficient lymphomas, RB was unable to promote senescence [92]. Similarly, using conditional oncogene K-*rasV12 *in a mouse model for human cancer initiation, Collada et al. [96] have shown that both in lung and in pancreas, premalignant tumor tissues displayed extensive senescent cells as indicated by the expression of different senescent markers including p16INK4a, p15INK4b, SA-β-gal, Dec1, DcR2 and SAHF, while these markers were rare or absent in the same tumors after they turned malignant; this could prove useful both in prognoss and diagnosis of cancer. Similarly, chemically induced premalignant skin papillomas with H-*Ras *oncogenic mutation displayed senescence marker *in vivo *[96].

Inactivation of the tumor suppressor PTEN (a lipid phosphatase that negatively regulates PI3 Kinase-AKT/PKB survival pathway) produces hyperplasticity in mice prostate epithelial cells similar to precancerous lesions in human prostate epithelium. Expression of senescence markers was reported [97] in these lesions, which was associated with inhibition of the development of malignancy. Absence of p53 prevents the senescence response to loss of PTEN and loss of both p53 and PTEN leads to invasive prostate carcinoma in mice [97]. In another study, using a conditional transgenic model with the mitogenic E2F3 transcription factor, Denchi et al., [98] have shown that E2F3 expression induced a burst of initial pituitary hyperplasia followed by a cessation of cell proliferation accompanied by expression of senescence markers. Michaloglou et al., [99] have reported that in cultures of human melanocytes and naevi, the benign precursors of malignant melanoma, an oncogenic allele BRAF, a protein kinase downstream of Ras, derived from human melanomas can induce sustained cell cycle arrest and senescence in fibroblasts and melanocytes, accompanied by the expression of p16INK4a and the common senescence marker, SA-β-gal. Congenital naevi *in vivo *were invariably positive for both SA-β-gal and spotty induction of p16INK4a expression, indicating that factors other than p16INK4a may cooperate with the mutant BRAF in bringing about senescence phenotype. The senescence phenotype was not brought about by telomere attrition, supporting the fact that oncogene-induced senescence is a genuine case of protective physiological process. After an initial cell proliferation, which results in the formation of naevi, such lesions typically remain static and benign.

Loss of Ku86 involved in chromosomal metabolism induces early onset of senescence in mice [100]. Telomere fusions responsible for breakage fusion bridge formation can be caused by mutations in the terminal region of telomeric DNA [101,102]. It has been recently shown that psychological stress, both perceived and chronic, is significantly associated with higher oxidative stress, lower telomerase activity, and shorter telomere length, which are known determinants of cell senescence and longevity, in peripheral blood mononuclear cells from healthy premenopausal women [103]

In general, the senescence checkpoint pathway genes such as MAPK [104,105], and overexpression of p53 [reviewed in [106]] and the genes that regulate p53 including ARF (p14^ARF ^in human or p19^ARF ^in mice), p33ING1 [107], PML [108-110], nucleoplasmin or NPM [111] and PTEN [97] are all involved in the senescence-induced tumor suppression program [Reviewed in [19,84]].

The above studies indicate that an initial burst of cell division due to activation of an oncogene expression in primary diploid cells results in the formation of premalignant tumor growth arrest with senescent morphology, and such lesions often remain benign without any further proliferation, until it progresses to a malignant state by additional mutations [112] and epimutations. This type of senescent phenotype appears to be caused by a telomere attrition-independent or proliferative history-independent mechanism and is termed premature or accelerated senescence which is likely to be favored by the absence of telomerase in somatic cells. However, cell types which maintain telomere length due to endogenous telomerase activity also display a senescent phenotype *in vitro*. An example is rodent fibroblasts with long telomeres and telomerase expression that can be induced to undergo premature senescence due to culture conditions [85] or after exposure to genotoxins [5]. Human epithelial cells when grown on plastic will undergo senescence, but, when cultured on a feeder layer, they proliferate indefinitely without any sign of senescence [113]. These data demonstrate that even in the absence of intrinsic mechanism of limited life span due to telomerase expression, such cells will respond to extrinsic factor(s)-induced cumulative damage to DNA by entering premature senescence phase.

### Induction of accelerated senescence as anti-cancer therapy

It is clear that the senescence phenotype can be induced under different conditions that might cause impediment to normal mitosis creating a mitotic crisis, including: (1) intrinsic ageing induced senescence (M1); (2) proliferative history dependent telomere attrition induced mitotic crisis (M2); (3) spontaneous, cumulative DNA damage induced senescence; (4) oncogene-induced accelerated senescence; and (5) genotoxin-induced premature senescence.

**Figure 1:**
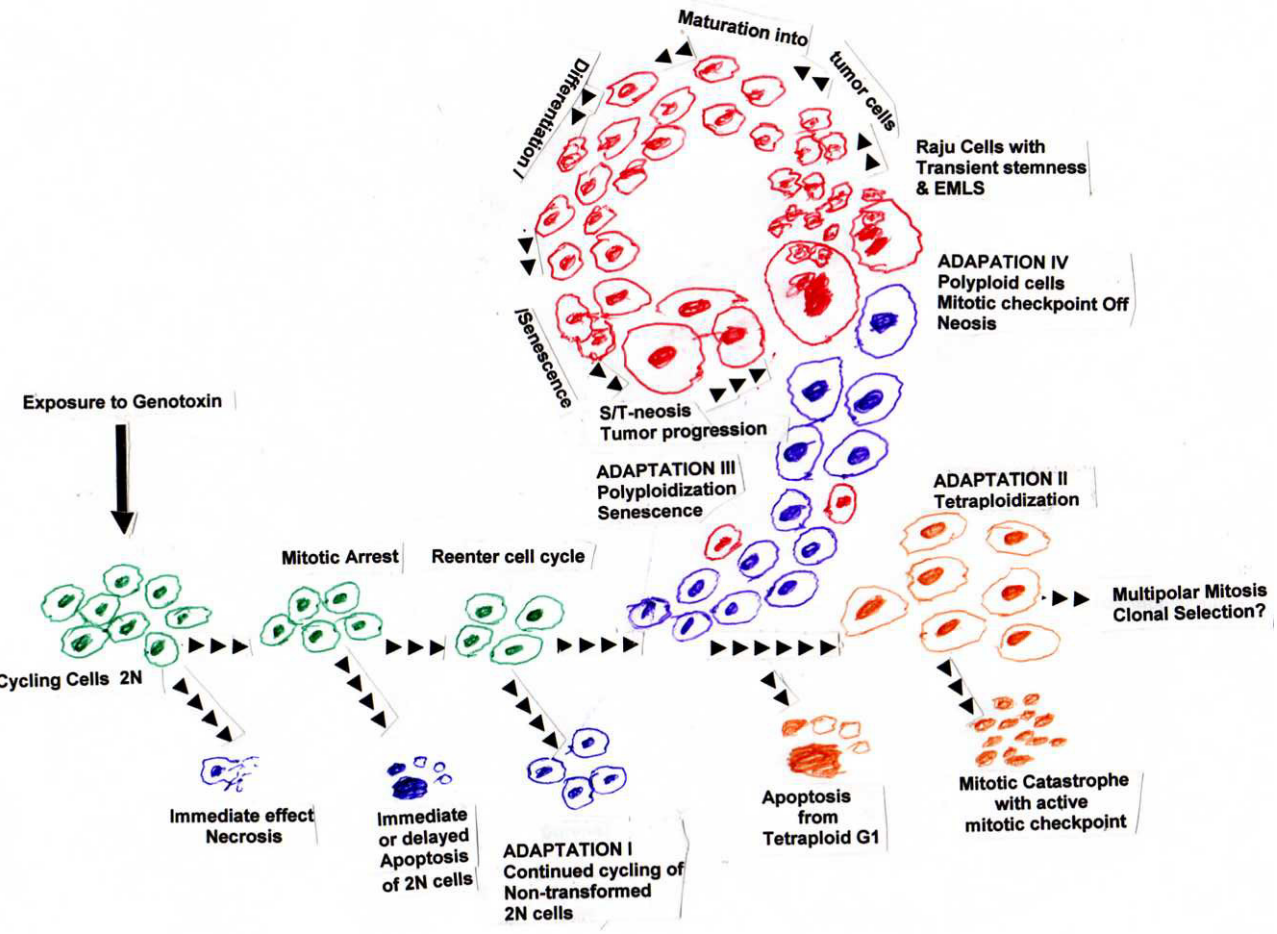
Fate of cells exposed to genotoxins: Immediate effect of exposure to genotoxins is the arrest of cell cycle progression. Cells with lethal damage will undergo necrotic death immediately or may commit immediate or delayed suicide by programmed cell death or apoptosis. Adaptation I. Some cells with minimal damage may re-enter cell cycle after some delay and repair of damage, and multiply normally without any immediate phenotypic changes. It is likely that some of these cells may carry epigenetic alterations and undergo neosis after a latent period of accumulation of additional damage to the genome. Adaptation Ii. Some cells become tetraploid due to cytokinesis failure. Some of them may commit apoptosis, or undergo mitotic catastrophe due to active mitotic checkpoint; such cells often form micronuclei during death. Some of them may undergo successfully multipolar mitosis, giving rise to aneuploid cells, which may not survive to give rise to clonal population of tumor cells. Adaptation III. A major fraction of cells enter a premature senescent phase due to genotoxin-induced DNA damage; by about a week or so, they express senescent markers such as SA-β-gal and SAHF in order to suppress tumor growth; they may become polyploid by endomitosis ad endoreduplication. Most of them may eventually die. Adaptation IV. By about second week after exposure to genotoxins, a few of the tetraploid and polyploid cells with genetic or epigenetic alterations in the senescence pathway may undergo neosis to give rise to aneuploid Raju cells with transient stemness. These are the precursors of primary tumor growth with extended MLS. They mature into tumor cells. At the end of their limited MLS, they reach senescent phase and undergo S/T-neosis and repeat the cycle of extended MLS, senescence, mitotic crisis and neosis several times, thus rejuvenating the supply of resistant (malignant) Raju cells in a highly non-synchronous fashion. (See the text for further details).

As described above, in primary cells, induction of an accelerated senescent phase acts as an efficient tumor suppressor mechanism; and DNA damaging agents or genotoxins also elicit an accelerated senescence-like phenotype [Reviewed in [114-116]] by activating DNA damage response pathways [88]. Tumor cells *in vivo *also display the phenomenon of senescence in response to exposure to genotoxins [reviewed in [19,84]]. Such cells may either undergo cytostasis and may never undergo mitosis again, even in the presence of growth factors, or they may die via apoptoss or mitotic catastrophe, Some cells may escape senescence and give rise to resistant tumor growth. (See Fig. 1 for the possible different fates of cells after exposure to genotoxins.)

Since senescence appears to be a tumor suppressor mechanism, it appears attractive to induce senescence in tumors *in vivo *in order to create a cytostatic state, where the tumor may not be completely eliminated, but can be maintained in a 'harmless' (non-proliferative) state [19,84]. This approach is especially attractive, since initiation of the senescent phenotype is accompanied by suppression of mitotic genes and overexpression of mitosis inhibitor genes [Reviewed in [84,117]]. Accordingly, te Poele et al., [118] have reported that in the archival breast tumors from patients who had undergone chemotherapy, when sectioned and stained for the senescent marker SA-β-gal, 41% of the tumors stained positive. In untreated controls only 10% of the tumors showed sporadic positive staining for SA-β-gal. Prof. E. Sikora, [Personal communication] has obsrved that SA-β-gal positive cells can undergo neosis, and tumor cells are apparently defective in senescent checkpoint control(s), and chances are that eventually some of these senescent cells might escape senescence and give rise to resistant tumor growth via neosis (see below). Therefore, induction of senescence as an anti-cancer therapy should be approached with caution for the following reasons:

1. Most of the genotoxins are carcinogens.

2. Tumor cells already have mutations in the senescent checkpoint pathway. Therefore, the chances of some cells escaping senescence are very high, especially in advanced tumors.

3. Senescent cells secrete factors that can promote tumor progression [81,119].

4. Senescent cells facilitate tumorigenesis in adjacent cells [119].

5. Ageing senescent cells may accumulate additional mutations due to oxidative damage and escape senescent phase via neosis and may result in recurrence of resistant tumor growth (See below).

6. Since most anti-cancer chemicals are carcinogenic, and the tumor tissue is a mixture of normal and tumor cells, chemotherapeutic drugs may facilitate tumorigenic transformation of normal or preneoplastic cells [19].

Therefore, the approach of controlling cancer by inducing senescence *in vivo*, although tempting, may in the long run increase the chances of resistant tumor growth, or facilitate origin of new tumors. One has to better understand the molecular events that regulate senescence and the mode of escape from senescence in tumors, the longevity and fate of senescent cells in vivo, before one can design effective anti-cancer treatment strategies based on senescence.

### What is neosis?

Neosis is the newly defined mode of cell division that occurs only in senescent, polyploid cells, and has not been observed in normal diploid cells. Neosis is a parasexual, somatic, reduction division displayed by a subset of multinucleate and/or polyploid giant cells or MN/PG cells formed during the spontaneous senescent phase of normal cells at the end of their MLS or genetic stress-induced accelerated senescence phase in tumor cells. It is characterized by: (1) chromosome distribution to daughter cells via nuclear budding in the presence of an intact nuclear envelope, (2) followed by asymmetric cytokinesis, giving rise to an indefinite number of small, aneuploid, mitotically active cells termed Raju cells (Raju meaning King in Telugu language), after which the polyploid neosis mother cell (NMC) dies. Raju cells display transient stem cell-like properties, and mature into tumor cells with extended MLS, finally arriving at a secondary/tertiary senescent phase. Neosis is interspersed with the extended MLS of Raju cells and their senescent phase and is repeated several times during tumor growth in a progressively non-synchronous fashion. Further, neosis is also responsible for the outgrowth of resistant tumor cells after exposure to genotoxins. Neosis appears to be a mode of origin and continuous growth of different tumor types, including hematological malignancies, carcinoma and sarcomas. The significant role of neosis appears to be fine tuning the damaged genome by epigenetic modulation in order to escape death via stress-induced senescence [5,6].

### Neosis-like events in the literature (Table 1)

**Table 1:** Neosis-like events reported in different cell systems of different species. N* = normal cells, T* = transformed or tumor cells, M* = Mutant cells.

Species	Cell type N* or T* 0r M*	MN/PGs	P- or S/T-neosis	Trigger	Conseuence	Reference
1. Snail	Primary cells N	Yes	P-neosis	Senescence	Established cell line	Walen, 2004
2. Chicken	Monocytes N	Yes	P-neosis	Senescence	Established cell line	Solari et al., 1965
3. Marsupial	Primary cells N	Yes	P-neosis	Senescence	Established cell line	Walen, 2004
4. Mouse	B16F10 melanoma cells	Yes	S/T-neosis (?)	Methotrexate	Resistant cell growth	Baroja et al. 1998.
5. Mouse	Embryonic stem cells M	Yes	P-neosis (?)	Parp-less	Teratocarcinoma	Nozaki et al., 1999
6. Mouse	C3H10T1/2 cells N	Yes	P-neosis	X-ray	Transformed foci	Sundaram et al. 2004
7. Mouse	C3H10T1/2 cells N	Yes	P-neosis	Etoposide	Transformed foci	Sundaram et al. 2004
8. Mouse	1ET1-C3H cells T	Yes	S/T-neosis	Spontaneous	Progression	Sundaram et al. 2004
9. Mouse	1ET1-C3H cells T	Yes	S/T-neosis	X-ray	Resistant cell growth	Sundaram et al 2004
10. Mouse	1ET1-C3H cells T	Yes	S/T-neosis	Etoposide	Resistant cell growth	Sundaram et al. 2004
11. Mouse	P53-/- MEF/MGB N	Yes	P-neosis	Senescence	Spont. Transformation	Sundaram et al. 2004
12. Mouse	P53+/+ MEF/MGB N	Yes	P-neosis	Senescence	Non-viable Raju cells	Sundaram et al. 2004
13. Mouse	P53-/- MEF/129B N	Yes	P-neosis	Senescence	Spont. Transformation	Sundaram et al. 2004
14. Mouse	P53+/+ MEF/129B N	Yes	P-neosis	Senescence	Non-viable Raju cells	Sundaram et al., 2004
15. Mouse	L cells T	Yes	S/T-neosis(?)	Arginine	Resistant tumor growth	Wheatley, Persnl communication
16. Armenian hamster	AHL cells N	Yes	P-neosis	X-ray	Transformed foci	Sundaram et al. 2004
17. Rat	REF N	Yes	P-neosis	X-ray	Transformed foci	Sundaram et al. 2004
18. Rat	X-REF23 N	Yes	P-neosis	X-ray	Transformed foci	Sundaram et al. 2004.
19. Rat	Adenocarcinoma cells	Yes	S/T-neosis	Cisplatin	Resistant tumor growth	Martin F, Persnl.communication
20. Mammals	Trophoblasts N	Yea	P-neosis (?)	Senescence	Non-viable Raju-like cells	Zybina et al., 1974, 1979
21. Human	Amniocytes N	Yes	P-neosis	Senescence	Estabished cell line	Zitcer and Dunnabecke, 1957
22. Human	HT 1080 cells T	Yes	S/T-Neosis	Chemical	Resistant tumor cells	Buikis et al., 1999
23. Human	Breast epithelial cells N	Yes	P-neosis (?)	Senescence	Transformed cell line	Romanov et al. q001.
24. Human	Prostate cancer cell PC3	Yes	S/t-Neosis	Doxotaxol	Resistant cell growth	Marakovskiy et al 2002
25. Human	Burkitt's lymphoma cells	Yes	S/T-neosis	Radiation	Resistant cell growth	Ivanov et al., 2003.
26. Human	Amnion cells N	Yes	P-neosis	senescence	Established cell line	Walen, 2004
27. Human	Amnion cells N	Yes	P-neosis	SV40	Transformed cells	Walen, 2004
28. Human	Adenocarcinoma cells	Yes	S/T-neosis	Spontaneous	Tumor progression	Sundaram et al. 2004
29. Human	FSK cells N	Yes	P-neosis	X-ray	Non-viable Raju cells	Sundaram et al. 2004
30. Human	MRC-5 cells N	Yes	P-neosis	X-ray	Non-viable Raju cells	Sundaram et al. 2004
31. Human	HTB11 cells T	Yes	S/T-neosis	Spontaneous	Tumor progression	Sundaram et al. 2004
32. Human	HTB11 cells T	Yes	S/T-neosis	X-ray	Tumor progression	Sundaram et al. 2004
33. Human	HeLa cells T	Yes	S/T-neosis	X-ray	Tumor progression	Sundaram et al. 2004
34. Human	Colon carcinoma HT116	Yes	S/T-neosis	Doxorubicin	Tumor progression	Sikora E, Persnl communication

Neosis-like events have been reported in the literature sporadically for more than a century [120] under different names [Reviewed in [6]]. Although to date, these events were not generally correlated with any functional significance, it is becoming clear that such a process is involved in the escape of senescent cells into neoplastic cells [Reviewed in [6]]. The earliest report that suggested a connection between neosis-like events with escape from the senescent primary cells was by Zitcer and Dunnabecke [121]. These authors reported that human senescent amniocytes bypassed senescence via nuclear budding followed by cellularization. Zybina et al., [122,123] have reported spontaneous polyploidization via endomitosis and endoreduplication during the differentiation of mammalian, extraembryonic placental trophoblast giant cells *in vivo*. Polyploidization was followed by fragmentation of the nucleus via nuclear budding into small individual aneuploid nuclei, resulting in synsytium-like post-budding multinucleate giant cells. Near the end of pregnancy, small individual cells were formed by asymmetric cytokinesis and cellularization. However, as in the case of p53+/+ MEF/MGB cells [5], these individual cells disintegrated without any further mitotic division. Based on the similarities between the steps involved in the maturation of trophoblasts and the process of neosis (Table 2), it was suggested [6] that gestational trophoblastic cancers [124,125] originate via neosis due to compromise of some tumor suppressor function at different stages of trophoblast development. It is apparent that such a phenomenon is involved in the origin of tumor cells, due to inadvertent expression of neotic gene(s) during DNA damage-induced loss of *p53 *or related senescent checkpoint genes.

**Table 2:** Similarities between trophoblast maturation and tumor cell self-renewal

Properties	Trophoblast	Tumor cell
1. Subject to ageing and senescence	Yes	Yes
2. Polyploidization by endomitosis and endoreduplication	Yes	Yes
3. Polyploid giant cell undergoes neosis	Yes	Yes
4. Activation of telomerase	Yes	Yes
5. Multiple neotic offspring	Yes	Yes
6. Degradation and migration through extracellular matrix	Yes	Yes
7. Secretion of proteases degrades extracellular matrix	Yes	Yes
8. Invasive properties	Yes	Yes
9. Proteolysis of thrombin receptor	Yes	Yes
10. Stimulation of invasive properties	Yes	Yes
11. Evasion of immune rejection	Yes	Yes
12. Activation of protooncogenes	Yes	Yes
13. Growth control by tumor suppressor genes	Yes. Under normal circumstances	No – Lost during neoplastic transformation
14. MLS of Raju cells or their equivalent	Limited MLS and perish at the end of pregnancy	Limited. Can extend MLS via repetitive S/T-neosis.

Solari et al., [126] have reported continuous production of small mononucleate Raju-like cells by nuclear budding from multinucleate giant polyploid cells formed by fusion of avian senescent peripheral blood mononuclear cells *in vitro*, even in the presence of an inhibitor of mitosis. These cells resembled Raju cells in their morphology and fused again initiating secondary nuclear budding to yield the next generation of Raju-like cells. Although the authors did not discus the relationship of their observations to senescence, immortalization or transformation of cells, these events are very similar to the primary (P-neosis) and secondary/tertiary or S/T-neosis reported by us [5,6]. Walen in a series of articles [127-129] has reported similar nuclear budding giving rise to viable cells with continuous division potential in different cell systems. Romanov et al., [130] have reported the immortalization of senescent human breast epithelial cells *in vitro*; although the exact events of this process has not been described; they mention a process of micronucleation, which could be the process of nuclear budding related to neosis [5] or the nuclear fragmentation that occurs during mitotic catastrophe [131].

While the above reports described the P-neosis-like events in aging primary cells, recently S/T-neosis-like events have been described in human cancer cell lines treated with genotoxns. Upon exposure of human tumor derived HT1080 cells to thiophosphomidium, cells reached a rapid senescence state and produced 'macro cells' (polyploid giant NMCs), which yielded small mononuclear cells (Raju cells), by nuclear budding, a process termed 'sporosis' [132]. In a detailed study on Burkitt's lymphoma cells with mutant p53, Erenpreisa and her co-workers [133-136] have described sequential events that occur during radiation-induced neosis-like events that resulted in the production of mitotically active Raju-like cells. Makaravskiy et al., [89] reported that exposure of PC3 prostate cancer cells to docetaxel resulted in growth arrest, multinucleation and giant cell formation, which gave rise to docetaxel-resistant clones; this resistance was associated with transient expression of a β-tubulin isoform and was independent of P-glycoprotein, bcl2 and bcl-xl expression. The recent literature showing neosis-like events are listed in Table 1. In every case, senescent MN/PGs yield mononuclear Raju-like cells that display extended MLS.

### Neosis bypasses senescence

In 1998, using computerized video time-lapse microscopy, we started to study the cellular process involved in transformed focus formation [5], which is considered to be the *in vitro *equivalent of *in vivo *tumorigenicity [70-72]. This involved: (1) video documentation of the transformation process at the level of individual cells, (2) isolation and cloning of the individual transformed foci without being contaminated by surrounding non-transformed cells, and (3) study the fate of these individual neotic clones (Raju cells derived from a single neosis mother cell or NMC) by serial cultivation under standard culture conditions and under anchorage independent conditions. These studies suggested that transformed focus formation occurs via the novel process termed neosis [5] and not via mitosis as previously thought.

Exposure of C3H10T1/2 cells to carcinogens induced accelerated formation of large senescent MN/PG cells that are supposed to be permanently arrested from undergoing mitosis and eventually die via mitotic catastrophe [19,81,137,138]. Around 14–21 days post-exposure to carcinogens, a minor subset of these senescence-like MN/PGs (hereafter termed the neosis mother cells or NMCs), before they died, produced several small mononuclear cells by karyokinesis via nuclear budding in the presence of the intact nuclear membrane. Each nuclear bud was immediately loaded with genomic DNA and surrounded by a small fragment of the cytoplasm delimited by plasma membrane by asymmetric cytokinesis. Each NMC produced about 10+/- 2 Raju cells. The latter were very small (~6–8 μm in diameter), had very high N/C ratio and immediately after birth, resumed symmetric mitotic division, inherited aneuploidy and genomic instability; they grew in soft agar, and displayed genotype and phenotype different from the mother cell. Presumably, they have reactivated telomerase expression [139-141] that resulted in the extension of their mitotic life span (MLS). Thus neosis and not mitosis is the mode of cell division resulting in tumorigenesis.

If neosis were the mode of transformation of normal cells into neoplastic cells, the spontaneous transformation of p53-/- mouse embryo fibroblasts should also involve senescent phase followed by neosis. In fact this is what we observed, when we studied the mode of spontaneous transformation of p53-/- cells using a similar approach. The primary cultures of p53-/- MEF/MGB cells entered senescent phase at the end of its diploid mitotic life span (about 6–7 passages), and almost all of these MN/PGs underwent spontaneous neotic division, each giving rise to 50 or more Raju cells, which by repetitive mitosis gave rise to spontaneously transformed cell lines. All the NMCs died within a month leaving the transformed cells in the Petri plate. The isogenic control wild type p53+/+ MEF/MGB cells underwent neosis yielding fewer number of Raju cells, but these Raju cells died without yielding a transformed cell line, probably due to the presence of p53, which is detrimental to cells with genomic instability [5,6,142] (see additional files 1, 2, 3, 4, 5, 6).

We also observed certain degree of plasticity in neosis, where the nuclear budding during karyokinesis may remain constant, but cytokinesis may follow immediately to form Raju cells sequentially or be delayed to form post-budding synsytium-like multinucleate cell, followed by cytokinesis yielding several Raju cells simultaneously [6].

### Raju cells transiently display stem cell properties

Raju cells are defined as the nascent daughter cells of neotic division, before they undergo their first symmetric mitosis. When the extended MLS of individual neotic clones was studied by serial subculturing, around the 20^th ^passage they spontaneously underwent a senescent phase and displayed mitotic crisis. Some of these MN/PGs spontaneously underwent secondary neosis, each NMC producing ~10 +/- 2 secondary Raju cells, only to repeat the cycle of EMLS followed by senescence, neosis, and production of the next generation of Raju cells. [5].

We and others have reported neosis occurring in several human and rodent tumor cell systems [5,6]. In tumor cell cultures, neosis was repeated several times each neotic division interspersed with extended, but limited, MLS of the neotic offspring followed by a senescent phase. Thus, during three years of continuous subculturing, human metastatic neuroblastoma HTB11 cells underwent three episodes of spontaneous senescence followed by S/T-neosis and yielded new populations of mitotically active Raju cells in a progressively non-synchronous fashion [5], (see additional files 1, 2, 3, 4, 5, 6). The first and second episodes of mitotic crisis lasted for about 7 to 8 weeks (with medium change twice a week) during which time the cells remained in the senescent phase. The crisis period was succeeded by neosis, giving rise to small, mononucleate Raju cells with extended MLS. However, when HTB11 cells were subjected to carcinogen exposure, the senescent phase lasted for only two weeks before the appearance of Raju cells, reducing the duration of senescent phase and mitotic crisis, indicating that genetic stress was high enough to induce neosis within a shorter duration. This implies that during spontaneous senescence and prolonged mitotic crisis (up to 7–8 weeks), the cells may slowly be accumulating additional mutations (probably due to oxidative stress) raising the level of genomic instability sufficient to induce spontaneous neosis.

We, therefore, hypothesize that self-renewal of cancer growth is made feasible by the transient re-expression of some stem cell properties in Raju cells. This, probably, occurs after epigenetic modulation of the non-viable polyploid genome of NMC prior to the neotic S phase or S_N _phase. Unlike in the mitotic S phase or S_M_, where DNA is replicated only once before each division, during the S_N _phase, DNA is replicated several times and the newly synthesized DNA is preferentially transported to the nuclear bud, followed by asymmetric cytokinesis. Thus, the NMCs display stem cell behavior in that the newly synthesized genomic DNA is asymmetrically segregated to the daughter Raju cells [5,6]. The nascent Raju cell genome often underwent mitotic division even before cellularization while still within the cytoplasm of the NMC [6,133].

Raju cells are unique in that they transiently display certain stem cell-like properties such as extended, but limited MLS, expression of telomerase, and potential to differentiate. They entered symmetric mitotic division immediately after they became independent cells. The mitotic derivatives of Raju cells grew larger in size and matured into tumor cells in a couple of days, while undergoing mitosis [5]. We interpret the increase in cell size during the course of mitotic division as an embryonic cell property that incorporates the G1 phase of the cell cycle [6,143]. As they undergo symmetric mitotic proliferation, they mature into tumor cells and probably undergo defective differentiation and gradually lose stem cell properties. At the end of their extended, but limited MLS, the tumor cells spontaneously entered a senescent phase. Since these cells have already lost some senescent checkpoint pathway gene(s) function, some of these senescent cells stand a good chance of undergoing S/T-neosis, thus contributing to the continuity of tumor cell lineage. Thus, Raju cells behave like committed stem cells immediately after birth and slowly acquire somatic cell properties during the prolipherative phase. Given in the box below are some of the stem cell properties of Raju cells and the somatic cell properties of their mitotic derivatives (Table 3).

**Table 3:** Properties of Raju cells and their mitotic derivatives:

**Transient stem cell properties of Raju cells:**
1. Short cell cycle duration of nascent Raju cells (before they undergo first mitosis) – an indication of lack of G1 phase? [5, 6].
2. Reactivation of telomerase conferring extended mitotic life span [139-141].
3. Is it possible to expand Raju cell population without differentiation under proper culture conditions such as EGF or FGF2 [43, 144-148].
4. Increase in cell size accompanied by increase in cell cycle duration-introduction of G1 phase in the cell cycle [Rajaraman, unpublished; 143].
5. Resistance to genotoxins – Expression of multidrug resistance genes? [192, 193].
6. Are they transiently expressing tissue stem cell specific surface markers? (e.g., CD34+ for hematopoietic cells [8]; CD133+ for brain cells [12, 13], CD44+, CD33-, LowLin- for breast cells [9-11]; CD20+ for skin cells [40]; CD44+,α 2β 1hi/CD133+ for prostate cancer cells [41].
7. Are they transiently expressing stem cell specific growth genes? (E.g. Nanog, Oct-4, Wnt, Bmi1 etc.) [188-191]
8. Potential to differentiate, although aberrantly.
**Somatic cell properties of mitotic derivatives of Raju cells:**
1. Resumption of symmetric mitotic division.
2. Increase in cell size – Introduction of G1 phase in the cell cycle? [143].
3. Progressive, but, aberrant differentiation.
4. Loss of tissue specific stem cell surface markers due to differentiation during extended mitotic proliferation?
5. Loss of expression of stem cell specific self-renewal genes?
6. Loss of expression of multidrug resistance genes?
7. They are subject to aging and associated senescence brought about by telomere attrition.
8. Therefore, they have limited division potential.
9. Telomere attrition, chromosome breakage-fusion-bridge cycle or genetic stress will result in senescent phase with MN/PG formation, mitotic crisis, and mitotic catastrophe.
10. Absence of senescent check points constitutes a built-in mechanism for accumulation of additional mutations via breakage-fusion-bridge cycle, setting in motion the next cycle of S/T-neosis [66, 67].

### Mitotic catastrophe and neosis are mutually exclusive with opposite effects

Chemotherapy and radiation therapy that target DNA as well as the process of cell division have been used with partial success as the main treatments of a variety of human tumors [144]. In normal cells, cell cycle checkpoints protect the cells from accumulating errors in the genome by blocking cells at different points in the cell cycle by enforcing the dependency of late events on the completion of early events [145]. Exposure of cells to genotoxins causes interruption of the progression of cell cycle due to these different cell cycle checkpoint controls. The individual cellular response to chemotherapeutic agents will depend upon the nature of the agent used, the position of the cell in the mitotic cell cycle, and the severity of the cellular damage. Cells with acute lethal damage may undergo immediate necrosis, or some of them will initiate the process of programmed cell death or apoptosis, which may lead to immediate cell suicide or delayed death by a day or two. In rare occasions, the cells may complete several mitotic divisions and the whole clone of cells might die simultaneously at a later date. Almost all the surviving cells will stop synthesizing DNA immediately. A major fraction of the surviving cells recover from the shock after several hours of quiescence and reenter cell cycle, probably after repair (or misrepair) of DNA damage. Most of such cells do not show any alterations in their morphology or growth behavior at least immediately after recovery [Fig. 1].

Of all the different checkpoint controls the most important one is the mitotic checkpoint or the spindle checkpoint, which is considered the primary defense against aneuploidy and ensures accurate chromosome segregation in order to produce genetically identical daughter cells [90,146]. During karyokinesis in mitosis and meiosis, the nuclear envelope is dismantled at which time the spindle checkpoint is activated [146,147]. Often the senescent cells form nuclear envelopes around fragments of the genome, a process termed micronucleation [131,148,149]. Thus, unable to maintain G2 arrest, they enter mitosis and after being arrested for several hours at metaphase, they eventually die without successfully completing mitosis. This process is known as mitotic catastrophe or mitotic death [150,151,154,155].

Since most cancer cells are deficient in the tumor suppressor function of the p53/pRB/p16INK4a signal transduction pathway, the G1, and G2 checkpoint functions are lost. After uncoupling apoptosis from G1 and S, tumor cells with mutant *p53 *arrive at G2/M interphase, at which time decisions regarding cell survival and death may be initiated. Cells that did not repair the damage to DNA arrive at G2 with point mutations and double strand breaks. Some of the double strand breaks are repaired by homologous recombination and non-homologous end rejoining during the G2 arrest [152,153]. Most of the surviving cells with unrepaired chromosomal lesions activate the senescent program via the DNA damage response. Senescent cells are characterized by SA-β-gal expression, SAHF, and are usually very large due to endomitosis and endoreduplication and are generally unable to divide again even in the presence of growth factors. Further, the senescent cells downregulate mitotic genes and upregulate anti-mitotic genes [19,81,84]. Under these circumstances, the senescent cancer cells will face inevitable death, unless they adapt and evade cell death, by eliminating the mitotically non-viable genome, and multiply by producing mitotically viable genome in order to be able to maintain the continuity of tumor cell lineage. All these conditions are fulfilled by neosis [5]. In this connection, it is interesting to note that senescent SA-β-gal positive cells formed after exposure of human colon carcinoma HT116 cells to doxorubicin underwent nuclear budding and formed Raju cells via neosis [Prof. E. Sikora, personal communication].

We postulated that during such critical time of mitotic crisis, the cells may revert back to neosis, which may be an evolutionary throw back just to tide over the crisis [5]. Interestingly, this mode of cell division resembles the asexual reproduction found in parasitic protozoans and protista and has been reported to be an evolutionary phase in sexual reproduction by meiosis in higher organisms [153]. However, it has been demonstrated that such a primitive mode of cell division is still being activated during the maturation of extraembryonic tropholast cells during mammalian pregnancy. Thus, it appears that the gene(s) that execute neosis are still functional in the mammalian genome, which are supposed to be active only during the trophoblast maturation in the extra embryonic tissue during pregnancy [122,123], and are inadvertently expressed in the absence of certain tumor suppressor genes (*p53*?) due to DNA damage.

When tumor cells are exposed to conventional chemotherapy or radiation therapy, most of the accelerated senescent cells may eventually die, but some do escape by adapting to the adverse conditions by undergoing polyploidization, and karyokinesis via nuclear budding [5,133]. Since mitotic checkpoint or spindle checkpoint is activated only after the dismantling of the nuclear envelope [146], polyploidization followed by nuclear budding with an intact nuclear envelope is bound to protect the NMC from cell death due to mitotic checkpoint. Therefore, some of these polyploid cells successfully undergo reduction division or de-polyploidization yielding near diploid daughter Raju cells with transient stem cell properties [5,6].

When spindle checkpoint is defective, a minor fraction of senescent cells escape mitotic catastrophe and become tetraploid due to mitotic slippage or cytokinesis failure [154,155]. Such cells have been shown to undergo multipolar mitosis giving rise to aneuploid cells [154]. However, it is not sure if these aneuploid cells will survive long enough to contribute to the clonal selection in tumor tissue [156]. We have shown that a subpopulation of tetraploid cells can undergo neosis by nuclear budding to give rise to several daughter cells before they die [5]. Since the nuclear envelope is not dismantled during neosis, spindle checkpoint is probably not activated and this might favor successful completion of neosis, often accidentally yielding aneuploid neotic offspring, which may survive and proliferate due to loss of genomic stability. During mitotic catastrophe, nuclear envelope is dismantled and spindle checkpoint is activated, leading to mitotic catastrophe via micronucleation and death [20,84,148-150]. Thus, mitotic catastrophe and neosis are mutually exclusive phenomena displayed by tumor cells with opposite effects. While the former eliminates the tumor cells with a defective genome, the latter helps adapt them to genomic instability-induced accumulation of genetic and epigenetic alterations by reducing the GI load in the neotic offspring via epigenetic modulation and thus contributing to the continuity of tumor cell lineage.

### Neosis and epigenetics in tumor progression

Epigenetic modulation is probably the way the genome alters its behavior in response to the environment [157]. "The genome functions like a highly sensitive organ of the cell that monitors its own activities and corrects common errors, senses unusual and unexpected events, and responds to them, often by restructuring itself" [158]. This statement probably accurately fits the behavior of cancer cells. Recent studies have revealed that genetic mutations alone do not lead to cancer and that epigenetics plays a major role in the origin and progression of tumors. Epigenetic alterations – non-DNA sequence-based heritable alterations – have been shown to initiate genomic instability, even before gene mutations enter into the process [159-162]. Epigenetic mutations fall into two main categories: (1) Altered DNA methylation of CpG dinucleotides, both losses or hypomethylation (results in gene activation) and gains or hypermethylation (results in silencing the gene) and (2) altered patterns of histone modifications such as acetylation or deacetylation of lysine residues, [160-166].

Epigenetic modulation occurs during all stages of tumor growth from the initiation at the progenitor cells through tumor formation and progression [162,163]. During the initiation stages, epigenetic modifications mimic the effect of genetic damage by altering the expression of tumor suppressor genes, thus compromising the tumor suppressor function of the senescence program; for example, silencing of tumor suppressor genes by promoter DNA hypermethylation and chromatin hypoacetylation, which may affect the expression of diverse genes including p53, RB1, p16INK4A, Von Hippel-Landau tumor suppressor (VHL) and MutL protein homologue 1 (MLH1) [109,162,163,168-170]. Global hypomethylation of chromatin and loss of imprinting lead to chromosomal instability and increased tumor incidence both *in vitro *and *in vivo *[165-167]; epigenetic activation of *R-ras *is responsible for gastric cancer [168] and cyclinD2 and mapsin activation in pancreatic cancer [169,170].

Tumor cells constitutively express some meiotic genes called Cancer/Testes (CT) antigens [171-174]. Immediately after exposure to radiation, some of these meiotic genes were translationally upregulated in Namalwa Burkitt's lymphoma cells with p53 mutant gene [175]. It has been recently demonstrated that exposure of tumor cells to genotoxins results in the translational upregulation of cMOS gene [175], which is involved in switching cells from mitosis to meiosis II and forcing the cell to undergo reduction division [172]. Constitutively expressed in low levels in untreated tumor cells, expression of meiotic cohesion gene REC8 was also enhanced after irradiation of p53 mutated Namalwa Burkitt's lymphoma cells, along with other meiosis-specific genes DMC1, STAG3, SYCP1 and SYCP3. Expression of these genes reached a peak level during the mitotic arrest phase and was proportional to the endopolyploid cells [175].

When such defective cells reach senescent phase and undergo neosis, these polyploid NMCs give rise to near-diploid or aneuploid Raju cells; this indicates that neosis must comprise properties of meiosis, mitosis and neosis specific events. During the extended MLS, Raju cells differentiate into tumor cells, while also accumulating additional mutational and epimutational alterations. This will increase the GI load, and cells will arrive at the next senescent phase due to incomplete differentiation displaying mitotic crisis. The onset of senescence is accompanied by several changes in the gene expression profile of the cells: (1) downregulation of mitotic genes; (2) upregulation of anti-mitotic genes [19,84]. During neosis the following changes in the gene-expression profile must occur: (1) reexpression of the 'immortalizing enzyme' telomerase is obligatory for the extension of mitotic life span, but not sufficient for neoplastic transformation [139-141,176,177]; (2) ectopic expression of meiotic genes collectively called cancer/testes (CT) antigens in tumor cells, which are thought to play a role in transformation [171-178], (3) in addition to ectopic expression of stem cell self-renewal genes including microRNAs, notch, oct4, and Bmi1 [77,179-184] and (4) multidrug resistance genes [185,186]. Therefore, the most significant event during neosis appears to be the alteration in gene expression profile, which is likely to be brought about by epigenetic modulation. This will in effect reduce the GI load, making it possible to produce mitotically viable genomes of Raju cells from the non-viable polyploid genome. Our data and those of others [5,6,127-129,152,153,175,187] suggest that the genome of such polyploid cells, although not mitotically viable, may undergo epigenetic modulation in order to reduce the degree of GI load to yield mitotically active daughter Raju cells with EMLS and thus contribute to the continuity of tumor cell lineage in a non-synchronous fashion, while the mitotically non-viable polyploid genome is eliminated by the post-neotic death of NMC. Thus, the genes for neosis which are silent in the genome, and should be active only during the maturation of the extraembryonic trophoblast cells during pregnancy [122,123], are reexpressed in tumor cells in the absence of p53 or related tumor suppressor genes due to DNA damage.

Neosis is repeated several times during the growth of tumors. This implies that global epigenetic modulation occurs throughout the life of the tumors, repetitively at least during each neosis, since continuous proliferation will lead to accumulation of gene mutations and epimutations, which may be often detrimental to the cell. Therefore, we propose that during neosis the cell with defective senescence checkpoint control(s) tend to undergo endoreplication/multinucleation and after restructuring the genome followed by multiple rounds of neotic S phase (S_N _phase), produces daughter Raju cells with EMLS via nuclear budding and asymmetric cytokinesis. This decreases the GI load in the Raju cells, while the non-viable polyploid genome of the NMC is discarded during its post-neotic demise. In the absence of tumor suppressor gene(s), the genome appears to be highly plastic (and tolerant to DNA damage) and responds to the damage and never activates the mitotic checkpoint by keeping the nuclear envelope intact and tides over the crisis by producing several Raju cells with stem cell properties and helps the continuous growth of tumor.

### 5.6. Neosis as the source of aneuploidy

The question of the source and mechanism of the origin of aneuploidy is still being debated [188]. More than a century ago, Hanesmann [189] and Boveri [120], suggested that aneuploidy is the result of non-disjunction during bipolar division or multipolor mitosis, respectively. The rare occurrence of pluripolar spindles represented Boveri's paradigm for a type of abnormal mitosis that can produce a variety of random chromosomal combinations. Unbalanced bipolar divisions or pluripolar mitoses will fail to distribute the chromosomal material to the daughter cells correctly. Therefore, both of these mechanisms can potentially give rise to tumor progenitors [120,189,190]. Accordingly, a parasexual cycle of polyploidization and segregation of chromosomes has been reported to occur in human fibroblasts, which has been assumed to involve multipolar spindle formation and chromosome non-disjunction [191,192]. However, it should be pointed out that the observations of both Hanesmann [189] and Boveri [120] were largely made in tumor cell populations, which led them to arrive at this conclusion. Since tumor cells have already lost genomic stability, they could tolerate errors in chromosomal distribution and continue dividing in order to survive [193]. Therefore, this does not address the question of origin of aneuploidy. We propose neosis as the third and most likely mode of arriving at aneuploidy for the following reasons:

(1) It has been shown recently that structural and numerical chromosome alterations in colon cancer develop through telomere-mediated anaphase bridges and not through mitotic multipolarity. In fact, multipolarity results in uneven chromosome distribution to daughter cells that gives rise to gross genomic changes such as nullisomies and non-viable daughter cells, and therefore, rarely contributed to clonal evolution tumor cells [194].

(2) It now seems that all human aneuploidies (cells that have chromosome number other than 46) in normal diploid human cell systems that occur during development result in embryonic lethality, except certain combinations of sex chromosomes and hyperdiploid, trisomies 13, 18 and 21, which yield severe birth defects in humans [195,196].

(3) Further, it has been recently shown that chromosome non-disjunction in primary human cells yields tetraploid cells rather than aneuploid cells due to failure of cytokinesis. This demonstrates that tetraploid cells do not directly give rise of aneuploidy. Therefore, there must be an intermediate step between tetraploid cells and the origin of aneuploid progeny of tumor cells [197].

(4) We observed primary neosis that gave rise to transformed cells in a binucleate giant cell with a chromosome bridge [5]. The fact that this NMC is binucleate with an isthmus, indicates that this cell has undergone one unsuccessful mitotic division attended by cytokinesis failure and has, therefore, an at least tetraploid genome.

(5) Thus, neosis appears to be the intermediate step between tetraploid cells and the origin of aneuploid tumor cells. It has the potential to give rise to aneuploid or near diploid stem cell-like Raju cells via polyploidization followed by karyokinesis via nuclear budding without activating mitotic checkpoint control by keeping the nuclear envelope intact.

(6) Accordingly, p53-/- MN/PG cells spontaneously gave rise to transformed cells via neosis [5] and in p53 null mouse cells cytokinesis failure-generated tetraploid cells promote tumorigenesis in vivo [198].

(7) These cells have extended MLS in the absence of senescent checkpoint controls (e.g. P53-/- cells); but they perish in the presence of proper checkpoint controls (eg., p53+/+ cells). Thus, p53+/+ MN/PG mouse cells did not yield viable Raju cells [5,6] and the p53+/+ tetraploid cells did not produce tumors *in vivo *[198].

(8) In support of this, it is also known that in several human and rodent tumor systems, tetraploidy is the intermediate stage before the genesis of neoplastic growth [155,156,199-203].

Therefore, we propose that senescent cells with tetraploid or higher ploidy genomes have the potential to under go neosis, creating conditions for automatic onset of aneuploidy to drive malignancy [67], if all the conditions for survival of the genome are met with in the resultant Raju cells.

Polyploidy can result either due to endomitosis and endoreduplication, or by cell fusion. It has been shown that Mad2- or BubR1-depleted cells (the genes involved in spindle checkpoint control) that do not complete cytokinesis remain viable through continued cycles of DNA replication up to at least 32 N [146,205]. In murine melanoma cultures exposed to methotrexate, emergence of resistant clones is preceded by an increase in polyploid cells with DNA content >8c and even >16c, concomitant with a decrease in tetraploid cells and is accompanied by loss of expression of mtotic proliferation markers (PCNA and CDK1) [206]. Even higher ploidy can be attained in certain cell systems [121-124,133]. Polyploidization, in addition to protecting cells from death confers evolvability by producing cells with non-lethal variations in the genome, upon which the process of natual selection may act [5,207,208]. In addition, since spindle checkpoint control is activated after the dissolution of the nuclear envelope [146], neosis, by performing karyokinesis via nuclear budding without the dissolution of the nuclear envelope, appears to be an adaptation by polyploid cells to escape from spindle checkpoint control-induced mitotic catastrophe. It is known that p53 null cells eventually develop polyploidy [5,156,197,198] attended by multiple centrosomes.

Often the presence of multiple centrosomes can avoid formation of multiple spindle poles by fusion of centrosomes resulting in a complex form of bipolar spindle [154,209]. However, it remains to be elucidated as to how the process of karyokinesis is effected in the presence of nuclear envelope. The question arises if the complex of multiple centrosomes acts like the spindle pole body of budding yeast by physically associating itself with the nuclear envelope and relocating itself back in the cytoplasm in Raju cells [129] so that they can resume symmetric mitosis.

Approximately one week after exposure to genotoxin, the chromatin in the endopolyploid giant cells reorganizes into a bouquet-like structure resembling meiotic prophase [132]. By about post-exposure day 14–21, some of these cells spontaneously undergo neosis and produce aneuploid Raju cells, the progenitors of tumor cells [5,127]. The latter undergo symmetric mitotic division and survive due to loss of checkpoint control(s) and gain of genomic instability. The role of meiotic genes in favoring reduction in the number of chromosomes during neosis is strongly suspected [175]. Similarly, multiple cycles of DNA replication in the NMC can be made possible by some meiotic gene(s) that are ectopically expressed in tumor cells [171-173][174]. Therefore, we suggest that, in addition to the global epigenetic modulation of the genome discussed above, neosis, rather than multipolar mitosis, is involved in the origin of aneuploid tumor cells.

However, we envisage that once the genesis of tumor cells via neosis is achieved, since these cells have gained genomic instability, further propagation of aneuploidy and the associated chromosomal aberrations including gene duplications, translocations etc will be facilitated by the various mechanisms including centrosome amplification, mitotic spindle abnormalities, defective attachment of chromatids to kinetchore, telomere dysfunction-induced breakage-fusion-bridge cycle, defective cytokinesis, mitotic checkpoint defects, among other things [188]. Thus, once the process of aneuploidy is initiated by neosis, progressive increase in aneuploidy might play an active role in the on-set of aggressive malignant property in solid tumors, whose survival and proliferation may be favored by loss of genomic stability [66].

The basis for the number of Raju cells produced by each NMC is not known. For example, in p53 MEF/MGB cells we observed that there were multiple giant nuclei present, and often more than one nucleus can produce nuclear buds and yield Raju cells. Number of Raju cells/NMC was around 10+/- 2 in C3H10T1 1/2 cells, ~4 or 5 in HeLa cells, and ~50 or more in p53-/-MGB/MEF cells, but 1 or 2 in p53-/-MEF/129B cells. While it is tempting to postulate that the number of Raju cells/NMC may depend upon the ploidy of the NMC, this remains to be tested. It is very likely in addition to ploidy, the cellular genetic background may be a determining factor in the number of Raju cells/NMC [5].

Additionally, it is known that cancer cells are living precariously at the edge of life [23]. While these cells have shut off spindle checkpoint and escaped mitotic catastrophe, and are on the way to escaping senescence, anything can go wrong in the process of neosis and cells may die at any stage of neosis, a process which we have termed neotic catastrophe. Thus, even at the last phase of neosis the NMC may die without yielding daughter cells, even after they have been successfully formed, for the simple reason that they cannot get out of the mother cells and become independent cells [5].

### Limits of extended Mitotic Life Span (MLS) and tumor progression

Neotic clones isolated from C3H10T1/2 cells exposed to etoposide displayed extended MLS, probably due to reexpression of telomerase [5,6,139-141]. Contrary to the belief that transformed cells are 'immortal,' we observed that at about the 20^th ^passage, the neotic clones entered the next cycle of senescence and underwent secondary neosis yielding the next batch of Raju cells, which continued to multiply [5,6]. Tumor cells undergo spontaneous senescence *in vivo *and display the senescence marker SA-β-gal sporadically [118] in a non-synchronous fashion. This implies that transformation does not confer immortality. Instead, this simply results in extension of the MLS of neotic offspring, which would not be feasible if it were not for the rejuvenation process of neosis. In addition, this observation suggests that the 'stemness' of Raju cells was transient and this property is lost during the proliferative phase, probably due to defective differentiation, which leads to the hierarchic nature of the tumor cell population. This important aspect of cancer cell mortality would not have been revealed if we did not study the behavior of individual neotic clones [5]. Under normal circumstances, the progenies of different NMCs will be growing either *in vitro *or *in vivo*, masking the repetitive renewal or rejuvenation of MLS via neosis, especially since this is occurring in a non-synchronized fashion.

Similarly, the human renal adenocarcinoma ACHN cells displayed rare MN/PG cells that initiated S/T-neosis; while in human metastatic neuroblastoma HTB11 cells, after continuous culture *in vitro *a high frequency of cells were observed to enter S/T-neosis [5]. Although we do not have any experimental data on the length of the extended MLS in these systems, the fact that these cells also display senescent MN/PGs that act as NMCs implies that cancer cells are not immortal, and that they also undergo spontaneous senescence due to differentiation and ageing [5] or accelerated senescence after exposure to anti-cancer agents [210]. However, the senescent cells may escape death since these are tumor cells with defective senescent check point control(s) and their growth is rejuvenated inconspicuously, since neosis is not synchronized.

Although there is limited knowledge about the genetics of life span and senescence, there are probably many genes involved in regulating these processes [211-213]. As the continuity of cancer cell lineage is facilitated by repetitive cycles of senescence followed by S/T-neosis and EMLS of tumor cells through cancer progression, there is increasing degree of non-synchrony in the onset of senescence. The senescence program is not intact in tumor cells. In addition, during the proliferative phase the tumor cells are known to accumulate additional random mutations increasing the degree of genomic instability load (GI load) [5,6,214]. It is likely that additional mutations or epimutations in the cancer cell genome would include the senescence genes, pro-apoptotic genes and genes that favor neosis. While such changes in the neosis-specific genes might have a negative selection effect, mutations or epigenetic changes in the senescent, pro-apopotic and longevity genes might have the opposite effect, contributing to the length of the extended MLS of tumor cells. However, one would expect an increase in the frequency of S/T-neosis due to the genetic stress caused by high GI load and accumulation of further mutational events in the genome. Additionally, tumor cells undergo defective differentiation, and this would also contribute to mitotic crisis along with the exhaustion of EMLS, leading to the next senescent phase. Thus, in malignant populations of cancer cells, one can expect to see a higher frequency of such cells may escape senescence via S/T-neosis. This is what we observed in the case of metastasizing neuroblastoma HTB11 cells [[5],; Rajaraman, unpublished]. It is tempting to suggest that in malignant tumor cell population, the NMCs may not display senescent markers such as SA-β-gal. The course of events that overcomes cellular senescence in human cancer pathology supports the above conclusion, even though the study did not involve neosis. For example, in the case of transitional cell carcinoma (TCC) that represents superficial bladder tumors and invasive bladder cancers, the superficial bladder tumor cells expressed p16 after limited *in vitro *passage and senesced as did the normal human uroepithelial cells, while all the muscle invasive TCCs contained altered p16 or pRB and bypassed senescence [215]. These data suggest that early tumors display senescence phase, while malignant tumors might have lost the efficiency of senescence barrier probably due to additional mutational events in genes that are involved in effecting the senescence program.

. When C3H10T2/3 mouse cells were exposed to 20 μM etoposide, the resultant neosis yielded Raju cell derivatives that were 25 times more resistant to etoposide; and they survived and underwent S/T-neosis even after exposure to 500 μM etoposide. The parent cell line died within a few days after exposure to 500 μM etoposide [5]. Prof. Martin reports that when rat colon adenocarcinoma cells were exposed to cisplatin, the resultant neotic progeny displayed resistance to genotoxins [Personal communication]. Makarovskiy et al. [89] have observed emergence of docetoxyl resistant prostate cancer PC3 cells after polyploidization. Erenpreisa and her coworkers have reported that when Burkitt's lymphoma cells were irradiated, the resultant neotic progenies were resistant to radiation [216]. Thus, neosis appears to be the primary source of epigenetic changes that fine tunes the damaged, non-viable polyploid genome to yield mitotically viable genome after global epigenetic modulation, and therefore, is the mechanism of recurrent growth of resistant tumor cells after chemotherapy or radiation therapy. It is proposed that when tumor cells are subject to adverse conditions *in vivo *or *in vitro*, cells undergo a rapid senescence phase and some of them may escape cell death by undergoing neosis and give rise to resistant tumor growth.

Up to ~10% of the cells in a given tumor tissue may be senescent cells (MN/PGs or potential NMCs). Senescent cells expressing SA-β-gal may undergo neosis [Dr. E. Sikora, personal communication]. At any given time, few of these potential NMCs in a tumor tissue will undergo S/T-neosis, thus replenishing the population of Raju cells, which will be subject to clonal selection. The individual neotic colonies are different from each other both phenotypically and genotypically [5]. Thus repetitive, non-synchronous neosis in tumor tissues constantly introduces heterogeneous populations of Raju cell derivatives, which will be subject to natural selection. Therefore, neosis is involved in maintaining the continuity of tumor cell lineage at times of mitotic crisis by producing resistant clones. This will result in the selection of progressively malignant cells, leading to tumor progression.

### Significance of neosis as a self-renewal mechanism

An unequivocal knowledge of the source of cancer cells and their mechanism of self-renewal have a special significance in developing effective anti-cancer therapeutic protocols, if one wants to kill cancer cells specifically without affecting the normal stem cells and somatic mitotic cells. Although the concept of cancer stem cell is very appealing (more than 31,000 articles have been published in support of this concept), it is still controversial [6,42,44]. In the absence of direct and compelling evidence for the asymmetric division potential of CSCs, and the recent emergence of evidence for the role of telomere attrition causing senescence in (adult) stem cells [52,53] and escape from senescence in the absence of senescence checkpoint control(s) [63], it is difficult to consider the concept of immortal CSCs as the source of cancer self-renewal.

On the other hand the use of transformed focus formation assay has demonstrated that neosis is not only the mode of origin of tumor cells with genomic instability, but also is an efficient mechanism of self-renewal that helps maintain the continuity of tumor cell lineage and is the mode of escape from senescence and mitotic catastrophe [[5,6] Prof. E Sikora, personal communication]. It is also involved in the origin of drug-resistant tumor growth [[5,6,89,132] Prof. F. Martin, personal communication]. Additionally, as discussed above, neosis appears to be the source of aneuploidy in tumor cells.

It is noteworthy that the NMCs resemble stem cells in that they undergo asymmetric division by segregating the newly synthesized viable genome to the daughter Raju cells, while retaining the non-viable defective polyploid genome, which is eliminated after the production of daughter Raju cells. The latter also display some degree of "stemness" in that they have gained extended MLS, indicating reactivation of telomerase; they have the potential to undergo differentiation, albeit aberrantly, and act as tumor initiating cells, contributing to the continuity of tumor cell lineage. Newer populations of mortal, but resistant (malignant) Raju cells with survival advantage due to transient expression of stem cell properties, which are repetitively produced via non-synchronous S/T-neosis due to selection pressure are operative in different tumor systems studied so far. This process appears to be a potent mode of self-renewal in tumor tissues. Taken together, these observations indicate that ESCs, ASCs and the transit amplifying cells, like the non-stem somatic cells [50,52,53,63,217] can also undergo senescent phase followed by extension of MLS, via neosis. This supports the alternative inference of the *in vivo *carcinogenic susceptibility data (see above) that the ASCs and their asymmetric mitotic progeny of transit amplifying cells, and not just the ASCs alone, are susceptible to carcinogen-induced mutational events (see above). In fact, the Raju cells with stem cell-like properties behave like committed stem cells or progenitor cells that lose their 'stemness' during their transit amplification and differentiation phase.

### 7. Cancers as a single disease of uncontrolled growth via neosis

Classically, cancer is considered a heterogeneous group of disorders with unlimited mitotic potential (immortal) and with markedly different biological properties, which are the result of clonal selection of mutant tumor suppressor genes and oncogenes. [218-222][223]. Thus, almost 200 different types of cancers, as many as the number of different types of cells in the human body, have been recognized, each with its own characteristic signal transduction pathways and with unique mutations in these differentiation pathways. Since each type of differentiated cell would reach its maturity via different signaling pathways, each type of cancer might have different molecular abnormalities that will be responsible for the uncontrolled growth of cancer [221]. This will be further complicated by the number of potential proto-oncogenes or tumor suppressor genes in the signal transduction pathways that can mutate or epimutate to cause cancerous growth. Further, the same gene may not carry an identical mutation even within a group of patients with one type of cancer [220]. This makes it very difficult for the modern approach of developing targeted therapy to treat cancers in a highly specific fashion for the individual cancer types and since the anticancer therapy is directed against mitotic tumor cells, in due course tumors become resistant to these drugs [224][225], probably, due to S/T-neosis-mediated emergence of resistant cells.

However, recent data, including ours suggest that tumor cell heterogeneity is due in part to epigenetic variation in the progenitor cells, and the epigenetic plasticity in addition to genetic variation is responsible to drive tumor progression [160,161]. This concept has added significance to ageing since even maternal twins display epigenetic differences as a function of their age and the environment they were brought up [164], This has resulted in great interest in the role of epigenetic mechanisms in the origin and progression of tumors [160-162].

Preliminary data indicate that neosis may be the common denominator for both solid tumors and hematological malignancies [5,6]. Up to 10% of the tumor cells are polyploid giant cells. Since these are the potential candidates for S/T-neosis to occur, effective elimination of these distinct populations of cells will reduce the chances of further progression of tumors. If one can successfully identify a common molecular step specific for neosis (see below for examples) among different cancer types, one can conceptualize cancer as a single disease caused by genesis and regenesis of Raju cells via neosis from the point of view of therapeutic molecular targeting. The common features of neosis in hematological malignancies, and solid tumors including carcinomas and sarcomas, may include DNA damage response-induced repair or misrepair, DNA polymerase(s) involved DNA repair and in polyploidization, epigenetic genome and chromatin modulation, activation of telomerase, DNA polymerase(s) involved in repetitive neotic DNA synthesis, karyokinesis via nuclear budding and asymmetric cytokinesis. This, hopefully, reduces the number of signal transduction pathways that can be altered during carcinogenesis in order to be able to interfere in the process of carcinogenesis. Thus design and development of an ideal anti-neotic agent or neociside to block the progression of multiple types of cancers may be simpler than the steps involved in identifying and developing molecular targets dependant on mitotic genes for individual cancer types or individual patients. Additionally, since senescent cells and therefore, neosis may not occur in normal somatic cells active in mitosis, the collateral damage to normal mitotic cells is bound to be highly reduced.

### Neosis paradigm of multistep carcinogenesis

Several theories of carcinogenesis have been proposed so far including: (1) the theory of step-wise accumulation of gain of function mutations in oncogenes and loss of function mutations in tumor suppressor genes [179]; loss of checkpoint control leading to genomic instability [2]; (3) aneuploidy-induced genomic instability [66]; (4) senescent checkpoint and telomere attrition [63,128,129]; (5) mutations in the apoptosis genes [126]; and (6) a combination of epigenetic and genetic alterations leading to genomic instability and tumorigenesis [160-164], among others.

The neosis paradigm of multistep carcinogenesis proposed by us encompasses all of the above phenomena, since the cancer cells seem to exploit these various phenomena during different stages of their evolution into malignancy. However, the major difference is that neosis and not mitosis is involved in the origin of tumors while tumor progression involves repetitive S/T-neosis interspersed with an extended, but limited, MLS and senescence between two neotic events [5,6]. Additionally, the common belief is that only stem cells are the progenitors of all cancers. The important property of a progenitor cell is its potential to proliferate. Even a fully differentiated cell can become cancerous, when infected by tumorigenic viruses, since these cancer causing viruses carry genes that can inactivate the tumor suppessor genes and promote cell division [78]. Therefore, neosis paradigm assumes that cells (ESCs, GSCs or ASCs) or progenitor cells at any stage of determination or differentiation pathway may be subject to mutational or epimutational changes. It is proposed that the mutational or epimutational damage should be sufficient to incapacitate mitotic division and to initiate the salvage pathway of neosis, without being lethal to the cell. In instances where the damage is not severe enough to inhibit successful completion of mitosis, the initiated damage will be fixed by promotion (cell proliferation) and P-neosis may be delayed until further accumulation of genetic damage during the proliferative phase [5], thus at least partially contributing to the latency of induced neoplasms [30,31]. P-neosis is preceded by genomic instability and followed by senescence and/or telomere attrition. Therefore, the resultant mitotic derivatives of Raju cells have the potential to become cancerous.

The following sequence of events and processes are envisaged to be exploited by cancer cells to survive and multiply in order to avoid death.

1. Accumulation of age-dependent epimutations [160-164] and both endogenous (inherited or through aging) and exogenous DNA damage may cause loss of checkpoint control(s) and genomic instability [63].

2. Telomere attrition may increase the GI load and induce the senescent phase and mitotic crisis [23,58,63].

3. Some cells escape from senescence by undergoing polyploidy, which confers survival value [5,6,207,208]. They still face cell death via mitotic catastrophe [20] and neotic catastrophe [5], while a few manage to rejuvenate the growth of cancer by producing neotic progeny with transient stem cell properties before they die.

4. In order to survive, the cell has to eliminate the mitotically non-viable polyploid genome and produce mitotically viable genome and multiply. Neosis fulfils all these requirements [5,6]. Delayed DNA repair [132,133,152,153] and epigenetic modulation [160,161,163] reduce the GI load [6] and the resultant viable daughter genome is copied several times and is asymmetrically distributed to the daughter Raju cells via nuclear budding and asymmetric cytokinesis [5,6]. Since the nuclear envelope is kept in tact during neosis, the spindle checkpoint is not activated and this protects the cells from death by mitotic catastrophe. The non-viable polyploid genome of the NMC is eliminated after producing several viable Raju cells [5].

5. Since the primary Raju cells have diploid or near diploid genomes derived from a non-viable polyploid genome [5,129,132,133], neosis constitutes a parasexual cycle of somatic reduction division, and probably incorporates some properties of meiosis and mitosis along with some unique properties of neosis [6,175].

6. The important properties of P-Raju cells (progeny of P-neosis) are genomic instability [2][5,129,227,228] and activation of telomerase or equivalent processes [63,64,139-141][228][229], meiotic genes [160-163], self-renewal genes of stem cells [181-184] and multidrug resistance genes [185,186].

7. Diploid or near diploid Raju cells of P-neosis with genomic instability have activated telomerase and diploid cells may inherit aneuploidy during the next S/T-neosis [6]. Introduction of aneuploidy creates an opportunity for breakage-fusion-bridge cycle giving rise to duplications, deletions and gene amplification and acts as the built in mechanism for an increase in GI load through extended mitotic proliferative phase [63,66,67].

8. Raju cells inherit lower GI load and with transient stem cell-like properties, display extended, but limited, MLS and are the tumor progenitors; their EMLS is subject to telomere attrition due to repetitive mitotic divisions, accompanied by defective differentiation. Therefore, their life span has to be rejuvenated by S/T-neosis.

9. Thus, tumor cells appear to be in fact mortal. Tumor cells will have to eventually die, if it were not for the process of neosis. Some senescent cells will attain polyploidy via endomitosis or endoreduplication, or by cell fusion and a subset of them will escape mitotic catastrophe via S/T-neosis, and regain extended MLS (Fig. 1).

10. Tumor cells are subjected to natural selection [68]. Each neotic division yields Raju cells resistant to the conditions at the time of neotic event [5,6,89,132,133,216].

11. Raju cells of advanced tumors may be different from those of the early or P-neosis, in that they may be more malignant, with altered MLS and may undergo non-synchronized S/T-neosis. Their MLS will be determined by the nature of the mutations in their senescent, pro-apototic, and longevity genes.

12. The primary event during neosis appears to be the epigenetic modification of the daughter cell genome resulting in fine tuning the gene expression profile to yield a mitotically viable cell from the mitotically nonviable polyploid genome, which is destined to be discarded soon after the successful completion of neosis.

13. Neosis helps cells evade death via mitotic catastrophe, and acts as a mechanism of cancer self-renewal by yielding resistant Raju cells with transient stem cell properties, and introduces heterogeneity in the tumor cell population for natural selection to act upon.

14. Thus, neosis is dynamically involved in maintaining the continuity of tumor cell lineage and tumor progression into malignancy. Non-synchronous occurrence of S/T-neosis creates the illusion of the existence of Cancer Stem Cells and the mirage of immortality of cancer cells.

### Future prospects

Although preliminary, over the past few years several laboratories both in Europe and in North America have reported neosis-like events in different normal and tumor cell types (Table 1). Studying the behavior of individual neotic clones has revealed the significance of their central role in cancer [5]. In almost all tumor cells *in vitro *and *in vivo*, MN/PG cells (potential NMCs) are found and at a given time, a minor population of NMCs will be undergoing neosis in a non-synchronous fashion. Therefore, a minor but variable fraction of Raju cells with transient stem cell-like properties will always be found in any tumor tissue, depending on the frequency of S/T-neosis. Based on our observations and those of others, we have provided preliminary evidence for a comprehensive and pivotal role for neosis in the origin and progression of tumors in different mammalian systems including human primary and metastatic tumor derived cell lines in vitro [5,6]. We have listed the properties of neosis as opposed to those of mitosis and meiosis [Table 2 in ref. [6]]. It is apparent that neosis is unique to the outgrowth of neoplastic cells from normal or established cell lines or tumor cells under conditions of high GI load, whether it is spontaneous, inherited or induced by chemical, viral or physical factors. The fact that MN/PG cells are ubiquitous in almost all types of cancers, both hematopoietic and solid tumors, suggests that common role for neosis in cancers is of great significance; but so far, did not receive the careful scrutiny it deserves.

NMCs and the process of neosis may be the Achilles' heel common to different types of cancers and therefore, may be cancer-specific targets to develop novel anti-neotic agents or "neosicides", which by definition could be effective in specific killing or inhibiting growth of different tumor types, without much non-specific side effects on normal cells. We suggest that it will be easier to target senescent NMCs rather than targeting actively dividing mitotic derivatives of Raju cells (equivalent of the so called cancer stem cells, but for the difference in their origin and fate?) in order to stop the growth of tumors. In addition, an ideal "neosicide" may be useful in preventing the on-set of primary tumor growth in high risk individuals. Although there is minimal information on the molecular events leading to and during neosis, the enormous data on hand concerning karyokinesis, cytokinesis, nuclear budding, cell cycle checkpoints, cyclins, oncogenes, tumor suppressor genes, genomic instability, apoptosis, mitotic catastrophe, and molecular profiles characterizing their alterations in different cancer types can be exploited to formulate a rational process for understanding the etiology of cancer and developing rational, effective and safe therapies against cancer.

## Conclusion

We trust that the above discussion has highlighted the drawbacks in the current concepts of the understanding of the biology of cancer pathogenesis. Contrary to the current belief, cancer originates via neosis and not via mitosis. Cancer cells are not immortal, since they also have limited MLS and are subject to senescence and some of them escape death by senescence due to mutations in the senescent checkpoint pathway. Finally, cancer self-renewal may not be because of immortal CSCs, but because of repetitive neosis that rejuvenates cancer growth by yielding newer populations of Raju cells, which are more malignant or resistant to the conditions that drove the neotic division.

Conventional non-surgical anti-cancer treatments such as chemotherapy and radiation therapy do not distinguish between mitotic normal and tumor cells and therefore, have proved to be not very effective at eradicating tumor growth and results in undesirable side effects. In addition, these therapies are known to induce the recurrent growth of resistant tumors probably via neosis in the place of the originally responsive tumors [5,6,131-133]. The discovery of neosis has identified novel cellular targets, against which one can identify novel neosis-specific molecular targets in order to design anti-neotic agents or neosicides. Further, since there are no senescent cells in the normal tissues where mitotic population is high, an ideal neosicide(s) is expected to be highly specific for tumor cells and is bound to minimize or eliminate undesirable side effects on normal mitotic cells. It is hoped that the above discussion of the significance of neosis in cancer biology will inspire further studies on this less traveled road on the way to better understanding cancer in order to help eliminate or minimize the human sufferings due to cancer.

## Abbreviations

ASCs, Adult or Tissue Stem cells; CSCs, Cancer Stem Cells; CT antigens, Cancer/Testes antigens; ESCs, Embryonic Stem Cells; HCS, hematopoietic stem cells, hMSCs, human mesenchymal stem cells; hTERT, human telomerase gene, MLS, Mitotic Life Span; EMLS, extended mitotic life span MN/PG, multinucleate/polyploid giant cells; NMC, Neosis Mother Cell.

## Availability

Additional files can also be found at [142]. All video clips are quicktime-Sorensen format with 15 frames/s. In VC 1–5, Images were recorded every 10 min and in VC.6 images were recorded every 5 min. For further details see Sundaram et al., 2004.

## Supplementary Material

Additional File 1 Primary neosis in a tetraploid MN/PG formed by C3H10T1/2 cells on post-irradiation day 14. Click here for file

Additional File 2 Neotic catastrophe in an MN/PG formed due to irradiation of C3H10T1/2 cells. Click here for file

Additional File 3 Spontaneous S/T-neosis in HTB11 cells. The first half of the clip shows a Raju cell emerging from a cell lying vertical on the centre right of the frame. The second half shows the emergence of a Raju cell from the cell lying horizontal in the centre of the frame. Click here for file

Additional File 4 Mitosis of a nascent Raju cell at the lower left of the frame. Click here for file

Additional File 5 Raju cell maturing into tumor cell with increase in cell mass displays clonogenecity. A group of Raju cells of varying ages are increasing in cell mass and undergoing mitosis giving rise to a colony of the next generation of tumor cells at the right half of the fame. Click here for file

Additional File 6 Spontaneous neosis in the p53-/-MEF/mgb cells. Click here for file
